# The genome sequence of the Common Buzzard, *Buteo buteo *(Linnaeus, 1758)

**DOI:** 10.12688/wellcomeopenres.23873.2

**Published:** 2025-09-26

**Authors:** Elizabeth Blows, Natural History Museum Genome Acquisition Lab, Darwin Tree of Life Barcoding collective, Wellcome Sanger Institute Tree of Life Management, Samples and Laboratory team, Wellcome Sanger Institute Scientific Operations: Sequencing Operations, Wellcome Sanger Institute Tree of Life Core Informatics team, Tree of Life Core Informatics collective, Darwin Tree of Life Consortium

**Affiliations:** 1 Raptor Foundation, Woodhurst, Cambridgeshire, England, UK

**Keywords:** Buteo buteo, Common Buzzard, genome sequence, chromosomal, Accipitriformes

## Abstract

We present a genome assembly from a male specimen of *Buteo buteo* (Common Buzzard; Chordata; Aves; Accipitriformes; Accipitridae). The assembly contains two haplotypes with total lengths of 1,303.68 megabases and 1,330.55 megabases. Most of haplotype 1 (94.6%) is scaffolded into 34 chromosomal pseudomolecules, including the Z sex chromosome. Haplotype 2 is a scaffold-level assembly. The mitochondrial genome has also been assembled, with a length of 18.36 kilobases.

## Species taxonomy

Eukaryota; Opisthokonta; Metazoa; Eumetazoa; Bilateria; Deuterostomia; Chordata; Craniata; Vertebrata; Gnathostomata; Teleostomi; Euteleostomi; Sarcopterygii; Dipnotetrapodomorpha; Tetrapoda; Amniota; Sauropsida; Sauria; Archelosauria; Archosauria; Dinosauria; Saurischia; Theropoda; Coelurosauria; Aves; Neognathae; Neoaves; Telluraves; Accipitrimorphae; Accipitriformes; Accipitridae; Accipitrinae; *Buteo*; *Buteo buteo* (Linnaeus, 1758) (NCBI:txid30397)

## Background

**Figure 1.**
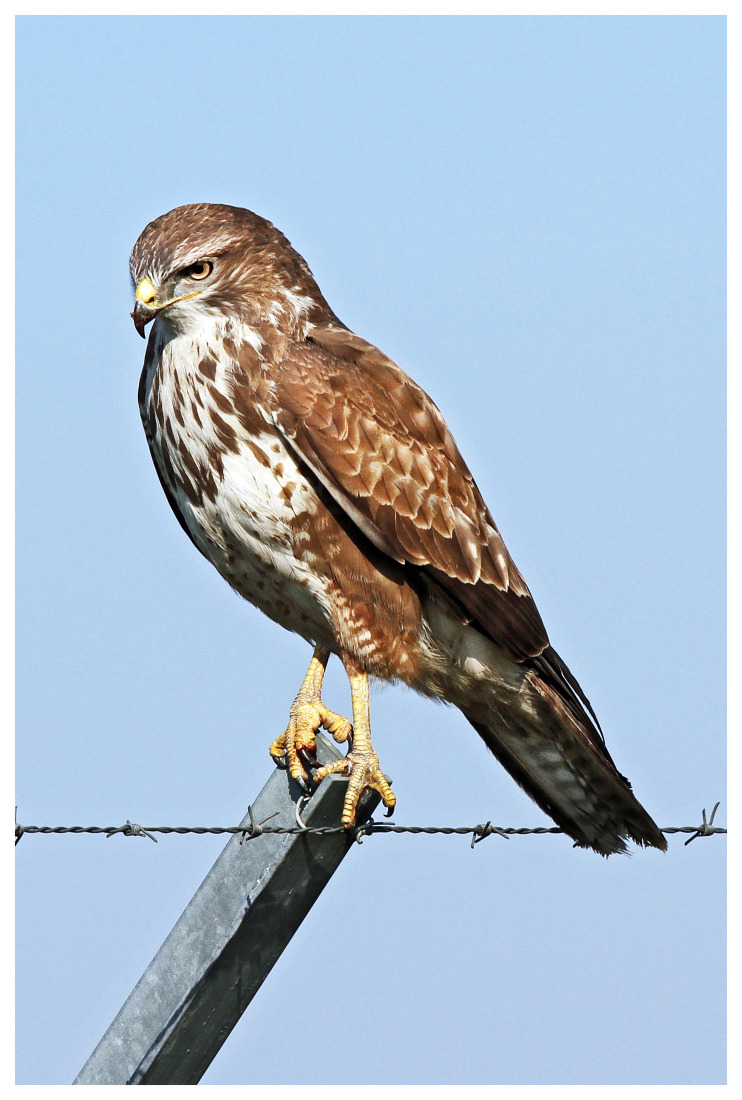
Photograph of *Buteo buteo* (not the specimen used for genome sequencing). Photograph by Björn Strey.

The common buzzard ( *Buteo buteo*) ( Figure 1) is the most widespread raptor in the UK ( Royal Society for the Protection of Birds (RSPB), no date; The Woodland Trust, no date) and across much of Europe and central Asia ( BirdLife International, 2020). It is a large bird of prey with broad, rounded wings and a short, fanned tail, often seen soaring with wings held in a shallow ‘V’. Plumage is highly variable, ranging from dark brown to much paler forms, but all individuals have dark wingtips and a finely striped tail. Its characteristic mewing call is often heard as it circles overhead.

Once severely reduced in numbers due to persecution, pesticide use, and declines in rabbit populations, buzzards have made a strong recovery in recent decades ( Border *et al.*, 2018). Legal protections, changes in land use, and reductions in pesticide use have allowed populations to expand significantly, particularly in eastern England, where they were previously absent ( Border *et al.*, 2018). Buzzards are now present in most rural landscapes, frequently seen perched on fences or lampposts or soaring over fields and woodlands ( Royal Society for the Protection of Birds (RSPB), no date; The Woodland Trust, no date). Despite this recovery, their densities and distribution are still influenced by habitat availability, prey abundance, and historical persecution pressures.

The genome of *Buteo buteo* was sequenced as part of the Darwin Tree of Life Project, a collaborative effort to sequence all named eukaryotic species in the Atlantic Archipelago of Britain and Ireland ( Blaxter *et al.*, 2022). Here we present a chromosome-level genome sequence for *Buteo buteo*, based on a male specimen from Abbots Ripton Road, Huntingdon, United Kingdom.

## Genome sequence report

### Sequencing data

The genome of a specimen of *Buteo buteo* was sequenced using Pacific Biosciences single-molecule HiFi long reads, generating 73.59 Gb (gigabases) from 7.11 million reads. GenomeScope analysis of the PacBio HiFi data estimated the haploid genome size at 1,265.45 Mb, with a heterozygosity of 0.24% and repeat content of 10.31%. These values provide an initial assessment of genome complexity and the challenges anticipated during assembly. Based on this estimated genome size, the sequencing data provided approximately 56.0x coverage of the genome. Chromosome conformation Hi-C data produced 78.18 Gb from 517.76 million reads. Table 1 summarises the specimen and sequencing information, including the BioProject, study name, BioSample numbers, and sequencing data for each technology.

**Table 1.** Specimen and sequencing data for *Buteo buteo*.

Project information			
**Study title**	Buteo buteo (common buzzard)		
**Umbrella BioProject**	PRJEB74952		
**Species**	*Buteo buteo*		
**BioSpecimen**	SAMEA114594453		
**NCBI taxonomy ID**	30397		
Specimen information			
**Technology**	**ToLID**	**BioSample accession**	**Organism part**
**PacBio long read** **sequencing**	bButBut1	SAMEA114594462	heart
**Hi-C sequencing**	bButBut1	SAMEA114594462	heart
**RNA sequencing**	bButBut1	SAMEA114594462	heart
Sequencing information			
**Platform**	**Run accession**	**Read count**	**Base count (Gb)**
**Hi-C Illumina NovaSeq X**	ERR12945459	5.18e+08	78.18
**PacBio Revio**	ERR12921306	7.11e+06	73.59
**RNA Illumina NovaSeq X**	ERR13493942	1.11e+08	16.81

### Assembly statistics

The genome was assembled into two haplotypes using Hi-C phasing. Haplotype 1 was curated to chromosome level, while Haplotype 2 was assembled to scaffold level. The assembly was improved by manual curation, which corrected 58 misjoins or missing joins. These interventions decreased the scaffold count by 1.09%. The final assembly has a total length of 1,303.68 Mb in 543 scaffolds, with 471 gaps, and a scaffold N50 of 46.96 Mb ( Table 2).

**Table 2.** Genome assembly data for *Buteo buteo*.

Genome assembly	Haplotype 1	Haplotype 2
Assembly name	bButBut1.hap1.1	bButBut1.hap2.1
Assembly accession	GCA_964188355.1	GCA_964188375.1
Assembly level	chromosome	scaffold
Span (Mb)	1,303.68	1,330.55
Number of contigs	1,014	929
Number of scaffolds	543	458
Assembly metrics* (benchmark)	Haplotype 1	Haplotype 2
Contig N50 length (≥ 1 Mb)	4.25 Mb	4.09 Mb
Scaffold N50 length (= chromosome N50)	46.96 Mb	44.78 Mb
Consensus quality (QV) (≥ 40)	64.5	64.9
*k*-mer completeness	94.20%	94.26%
Combined *k*-mer completeness (≥ 95%)	99.82%	
BUSCO** (S > 90%; D < 5%)	C:97.3%[S:97.0%,D:0.3%],F:0.6%, M:2.1%,n:8338	-
Percentage of assembly mapped to chromosomes (≥ 90%)	94.6%	Scaffold level
Sex chromosomes (localised homologous pairs)	Z	-
Organelles (one complete allele)	Mitochondrial genome: 18.36 kb	-

* BUSCO scores based on the aves_odb10 BUSCO set using version 5.5.0. C = complete [S = single copy, D = duplicated], F = fragmented, M = missing, n = number of orthologues in comparison.

**Figure 2.**
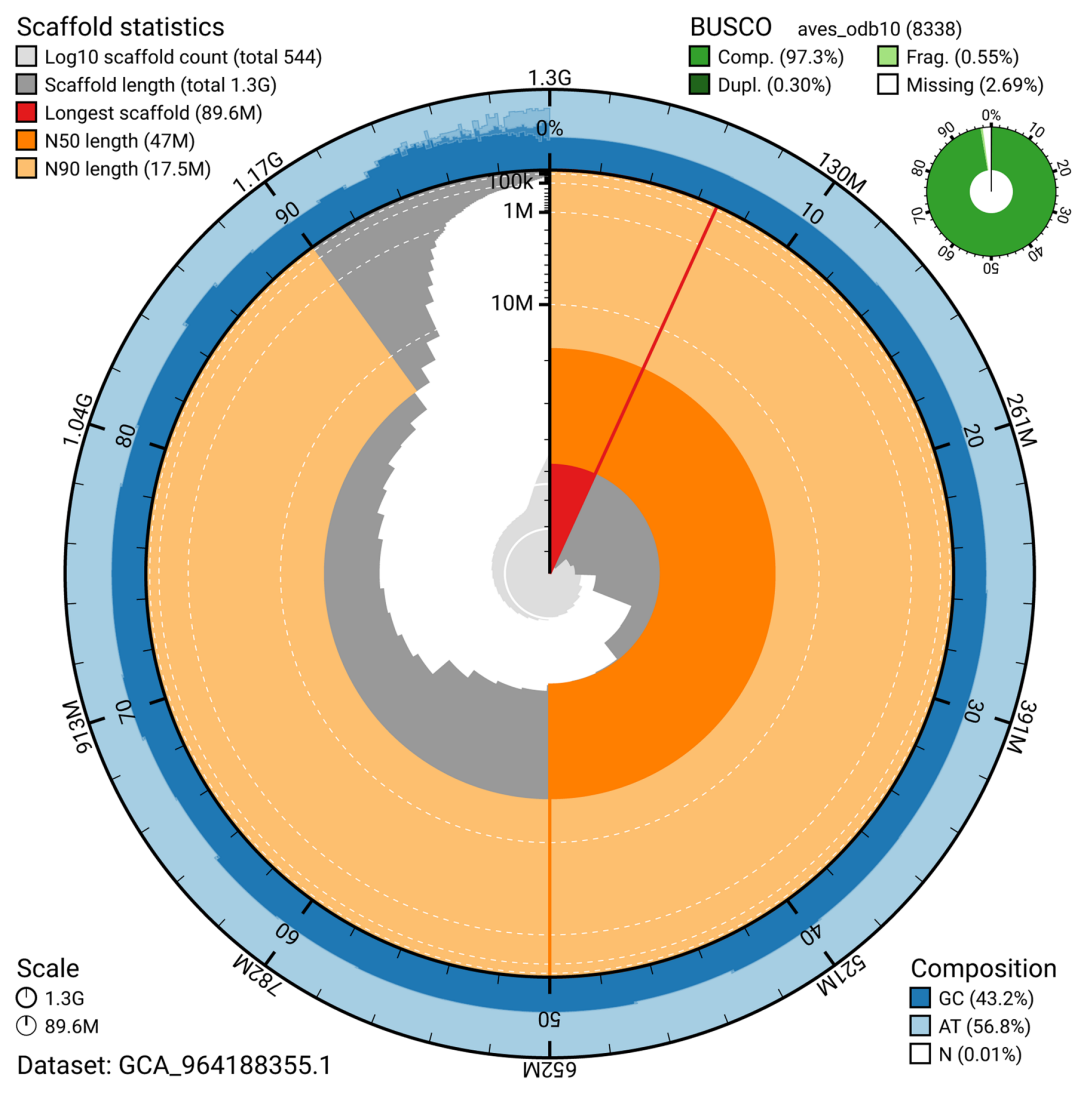
Genome assembly of *Buteo buteo*, bButBut1.hap1.1: metrics. The BlobToolKit snail plot provides an overview of assembly metrics and BUSCO gene completeness. The circumference represents the length of the whole genome sequence, and the main plot is divided into 1,000 bins around the circumference. The outermost blue tracks display the distribution of GC, AT, and N percentages across the bins. Scaffolds are arranged clockwise from longest to shortest and are depicted in dark grey. The longest scaffold is indicated by the red arc, and the deeper orange and pale orange arcs represent the N50 and N90 lengths. A light grey spiral at the centre shows the cumulative scaffold count on a logarithmic scale. A summary of complete, fragmented, duplicated, and missing BUSCO genes in the aves_odb10 set is presented at the top right. An interactive version of this figure is available at https://blobtoolkit.genomehubs.org/view/GCA_964188355.1/dataset/GCA_964188355.1/snail.

**Figure 3.**
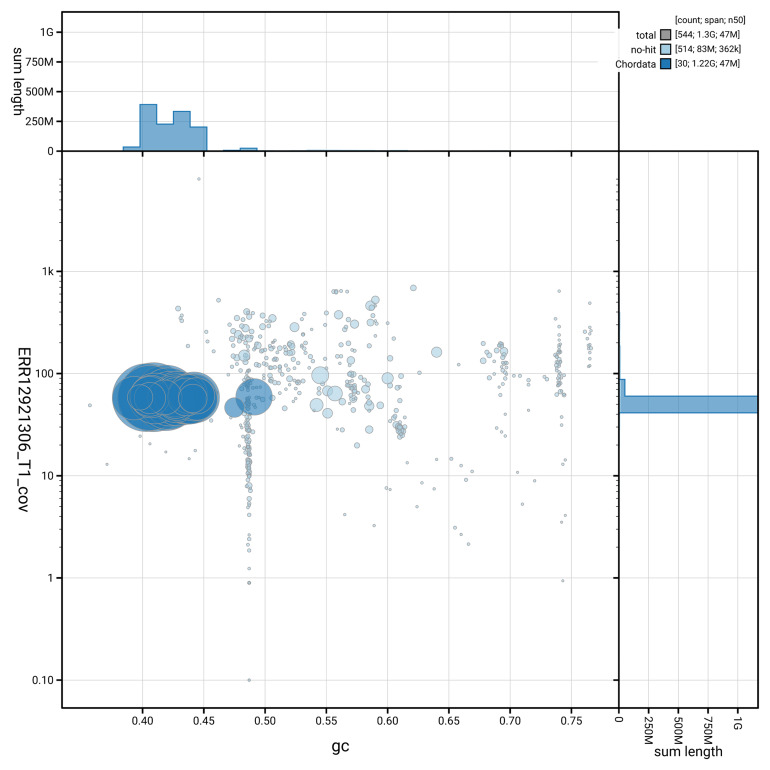
Genome assembly of *Buteo buteo*, bButBut1.hap1.1: BlobToolKit GC-coverage plot. Blob plot showing sequence coverage (vertical axis) and GC content (horizontal axis). The circles represent scaffolds, with the size proportional to scaffold length and the colour representing phylum membership. The histograms along the axes display the total length of sequences distributed across different levels of coverage and GC content. An interactive version of this figure is available at https://blobtoolkit.genomehubs.org/view/GCA_964188355.1/dataset/GCA_964188355.1/blob.

**Figure 4.**
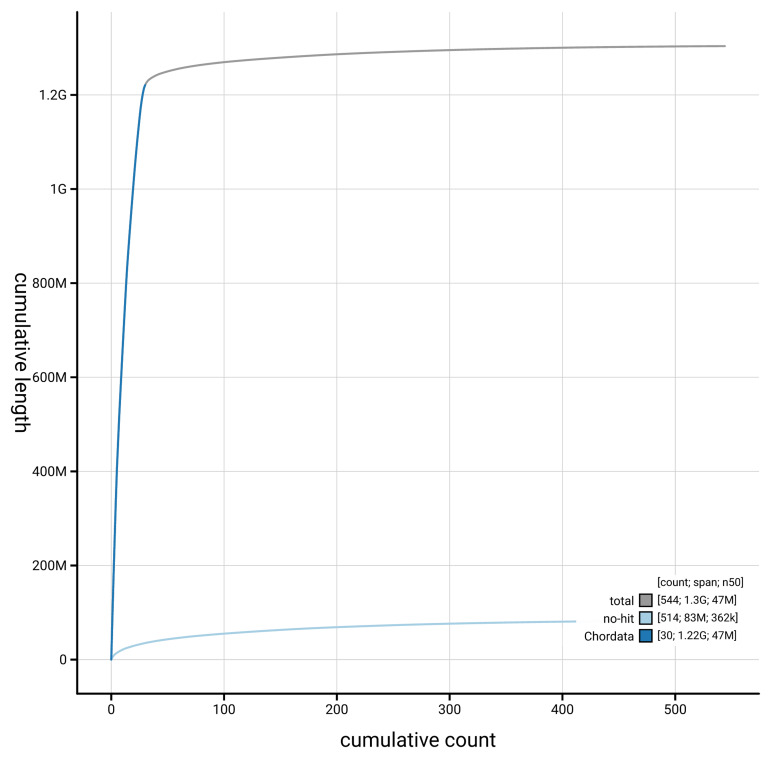
Genome assembly of *Buteo buteo,* bButBut1.hap1.1: BlobToolKit cumulative sequence plot. The grey line shows cumulative length for all scaffolds. Coloured lines show cumulative lengths of scaffolds assigned to each phylum using the buscogenes taxrule. An interactive version of this figure is available at https://blobtoolkit.genomehubs.org/view/GCA_964188355.1/dataset/GCA_964188355.1/cumulative.

The snail plot in Figure 2 provides a summary of the assembly statistics, indicating the distribution of scaffold lengths and other assembly metrics. Figure 3 shows the distribution of scaffolds by GC proportion and coverage. Figure 4 presents a cumulative assembly plot, with separate curves representing different scaffold subsets assigned to various phyla, illustrating the completeness of the assembly.

**Figure 5.**
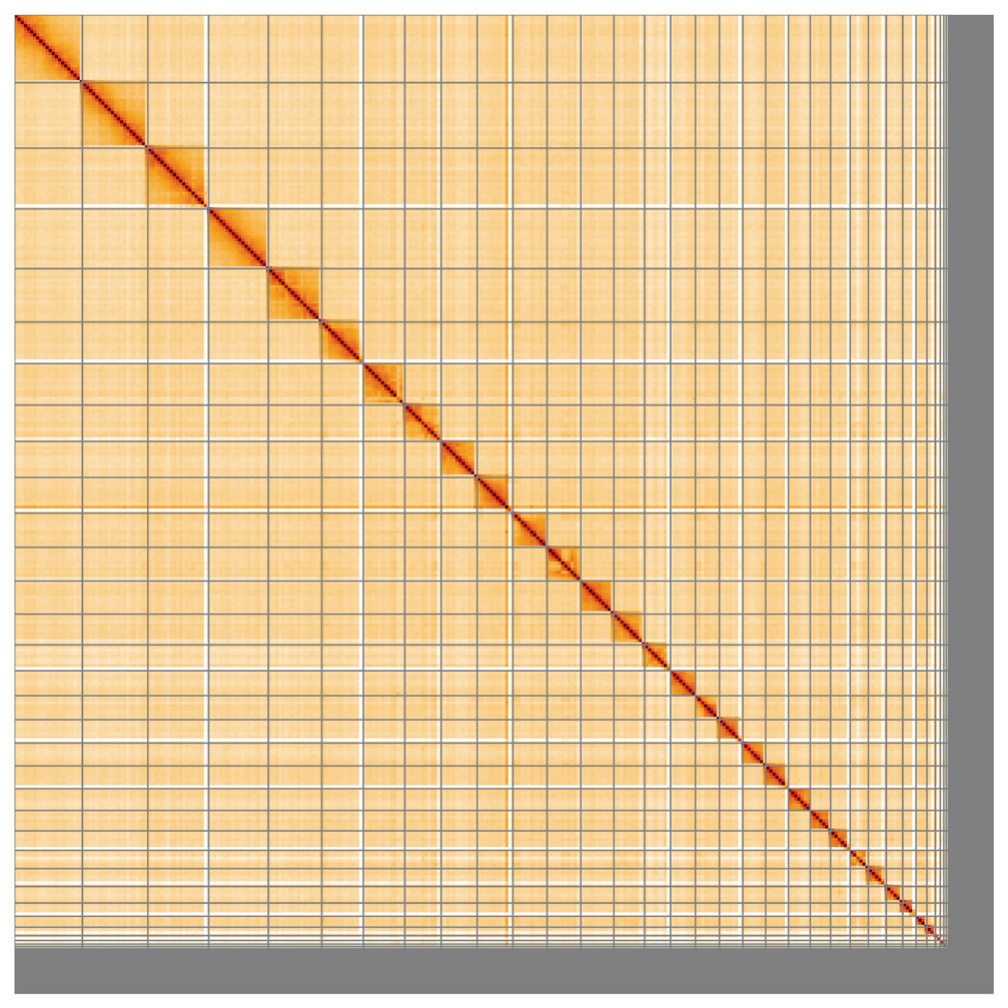
Genome assembly of *Buteo buteo*: Hi-C contact map of the bButBut1.hap1.1 assembly, visualised using HiGlass. Chromosomes are shown in order of size from left to right and top to bottom. An interactive version of this figure may be viewed at https://genome-note-higlass.tol.sanger.ac.uk/l/?d=NJh5Uze1TWmCv27O2QITmA.

Most of the assembly sequence (94.6%) was assigned to 34 chromosomal-level scaffolds, representing 33 autosomes and the Z sex chromosome. These chromosome-level scaffolds, confirmed by Hi-C data, are named according to size ( Figure 5; Table 3). During curation, it was noted that the sample is the homogametic sex, ZZ (male). Chromosome Z was identified and assigned by synteny to *Gypaetus barbatus* (GCA_028022735.1).

**Table 3.** Chromosomal pseudomolecules in the genome assembly of *Buteo buteo*, bButBut1.

INSDC accession	Name	Length (Mb)	GC%
OZ076920.1	1	86.32	40.5
OZ076921.1	2	80.24	42
OZ076922.1	3	78.77	40.5
OZ076923.1	4	70.32	42
OZ076924.1	5	54.89	42
OZ076925.1	6	54.75	43
OZ076926.1	7	47.85	44
OZ076927.1	8	47.55	41
OZ076928.1	9	46.96	43.5
OZ076929.1	10	45.13	43.5
OZ076930.1	11	44.56	44
OZ076931.1	12	43.48	42.5
OZ076932.1	13	40.5	44
OZ076933.1	14	34.27	39.5
OZ076934.1	15	32.75	40.5
OZ076935.1	16	31.57	44.5
OZ076936.1	17	31.02	43
OZ076937.1	18	30.04	42.5
OZ076938.1	19	30.01	42.5
OZ076939.1	20	28.73	40.5
OZ076940.1	21	26.9	43.5
OZ076941.1	22	25.81	43.5
OZ076942.1	23	23.95	49
OZ076943.1	24	23.6	44.5
OZ076944.1	25	21.76	42
OZ076945.1	26	17.46	40.5
OZ076946.1	27	14.37	44
OZ076947.1	28	11.12	40
OZ076948.1	29	6.43	47.5
OZ076949.1	30	4.7	54.5
OZ076950.1	31	3.44	55.5
OZ076951.1	32	2.6	54
OZ076952.1	33	1.85	48.5
OZ076919.1	Z	89.59	41
OZ076953.1	MT	0.02	45

The mitochondrial genome was also assembled. This sequence is included as a contig in the multifasta file of the genome submission and as a standalone record in GenBank.

### Assembly quality metrics

The estimated Quality Value (QV) and *k*-mer completeness metrics, along with BUSCO completeness scores, were calculated for each haplotype and the combined assembly. The QV reflects the base-level accuracy of the assembly, while *k*-mer completeness indicates the proportion of expected *k*-mers identified in the assembly. BUSCO scores provide a measure of completeness based on benchmarking universal single-copy orthologues.

For haplotype 1, the estimated QV is 64.5, and for haplotype 2, the QV is 64.9. When the two haplotypes are combined, the assembly achieves an estimated QV of 64.7. The *k*-mer completeness for haplotype 1 is 94.20%, and for haplotype 2 it is 94.26%. When the two haplotypes are combined, the assembly achieves a *k*-mer completeness of 99.82%. BUSCO 5.5.0 analysis using the aves_odb10 reference set ( *n* = 8,338) achieved a completeness score of 97.3% (single = 97.0%, duplicated = 0.3%) for haplotype 1.

Table 2 provides assembly metric benchmarks adapted from Rhie *et al.* (2021) and the Earth BioGenome Project (EBP) Report on Assembly Standards September 2024. The assembly achieves the EBP reference standard of **6.C.Q64.**

## Methods

### Sample acquisition

An adult male *Buteo buteo* (specimen ID NHMUK014446389, ToLID bButBut1) was collected from Abbots Ripton Road, Huntingdon, United Kingdom (latitude 52.35, longitude –0.18) on 2021-01-10. The bird was treated for injuries at the Raptor Foundation, but died within a few hours of arrival. The specimen was collected and identified by Liz Blows (Raptor Foundation) and samples were preserved by dry freezing (–80 °C). Metadata collection for samples adhered to the Darwin Tree of Life project standards described by Lawniczak *et al.* (2022)

### Nucleic acid extraction

The workflow for high molecular weight (HMW) DNA extraction at the Wellcome Sanger Institute (WSI) Tree of Life Core Laboratory includes a sequence of procedures: sample preparation and homogenisation, DNA extraction, fragmentation and purification. Detailed protocols are available on protocols.io ( Denton *et al.*, 2023b).

The bButBut1 sample was prepared for DNA extraction by weighing and dissecting it on dry ice ( Jay *et al.*, 2023). Tissue from the heart was homogenised using a PowerMasher II tissue disruptor ( Denton *et al.*, 2023a). HMW DNA was extracted using the Automated MagAttract v2 protocol ( Oatley *et al.*, 2023a). DNA was sheared into an average fragment size of 12–20 kb in a Megaruptor 3 system ( Bates *et al.*, 2023). Sheared DNA was purified by solid-phase reversible immobilisation, using AMPure PB beads to eliminate shorter fragments and concentrate the DNA ( Oatley *et al.*, 2023b). The concentration of the sheared and purified DNA was assessed using a Nanodrop spectrophotometer and Qubit Fluorometer using the Qubit dsDNA High Sensitivity Assay kit. Fragment size distribution was evaluated by running the sample on the FemtoPulse system.

RNA was extracted from heart tissue of bButBut1 in the Tree of Life Laboratory at the WSI using the RNA Extraction: Automated MagMax™ *mir*Vana protocol ( do Amaral *et al.*, 2023). The RNA concentration was assessed using a Nanodrop spectrophotometer and a Qubit Fluorometer using the Qubit RNA Broad-Range Assay kit. Analysis of the integrity of the RNA was done using the Agilent RNA 6000 Pico Kit and Eukaryotic Total RNA assay.

### Hi-C sample preparation and cross-linking

Tissue from the heart of the bButBut1 sample was processed for Hi-C sequencing at the WSI Scientific Operations core, using the Arima-HiC v2 kit. In brief, 20–50 mg of frozen tissue (stored at –80 °C) was fixed, and the DNA crosslinked using a TC buffer with 22% formaldehyde concentration. After crosslinking, the tissue was homogenised using the Diagnocine Power Masher-II and BioMasher-II tubes and pestles. Following the Arima-HiC v2 kit manufacturer's instructions, crosslinked DNA was digested using a restriction enzyme master mix. The 5’-overhangs were filled in and labelled with biotinylated nucleotides and proximally ligated. An overnight incubation was carried out for enzymes to digest remaining proteins and for crosslinks to reverse. A clean up was performed with SPRIselect beads prior to library preparation. Additionally, the biotinylation percentage was estimated using the Qubit Fluorometer v4.0 (Thermo Fisher Scientific) and Qubit HS Assay Kit and Arima-HiC v2 QC beads.

### Library preparation and sequencing

Library preparation and sequencing were performed at the WSI Scientific Operations core.

***PacBio HiFi***

At a minimum, samples were required to have an average fragment size exceeding 8 kb and a total mass over 400 ng to proceed to the low input SMRTbell Prep Kit 3.0 protocol (Pacific Biosciences, California, USA), depending on genome size and sequencing depth required. Libraries were prepared using the SMRTbell Prep Kit 3.0 (Pacific Biosciences, California, USA) as per the manufacturer's instructions. The kit includes the reagents required for end repair/A-tailing, adapter ligation, post-ligation SMRTbell bead cleanup, and nuclease treatment. Following the manufacturer’s instructions, size selection and clean up was carried out using diluted AMPure PB beads (Pacific Biosciences, California, USA). DNA concentration was quantified using the Qubit Fluorometer v4.0 (Thermo Fisher Scientific) with Qubit 1X dsDNA HS assay kit and the final library fragment size analysis was carried out using the Agilent Femto Pulse Automated Pulsed Field CE Instrument (Agilent Technologies) and gDNA 55kb BAC analysis kit.

Samples were sequenced on a Revio instrument (Pacific Biosciences, California, USA). Prepared libraries were normalised to 2 nM, and 15 μL was used for making complexes. Primers were annealed and polymerases were hybridised to create circularised complexes according to manufacturer’s instructions. The complexes were purified with the 1.2X clean up with SMRTbell beads. The purified complexes were then diluted to the Revio loading concentration (in the range 200–300 pM), and spiked with a Revio sequencing internal control. Samples were sequenced on Revio 25M SMRT cells (Pacific Biosciences, California, USA). The SMRT link software, a PacBio web-based end-to-end workflow manager, was used to set-up and monitor the run, as well as perform primary and secondary analysis of the data upon completion.

***Hi-C***

For Hi-C library preparation, DNA was fragmented using the Covaris E220 sonicator (Covaris) and size selected using SPRISelect beads to 400 to 600 bp. The DNA was then enriched using the Arima-HiC v2 kit Enrichment beads. Using the NEBNext Ultra II DNA Library Prep Kit (New England Biolabs) for end repair, a-tailing, and adapter ligation. This uses a custom protocol which resembles the standard NEBNext Ultra II DNA Library Prep protocol but where library preparation occurs while DNA is bound to the Enrichment beads. For library amplification, 10 to 16 PCR cycles were required, determined by the sample biotinylation percentage. The Hi-C sequencing was performed using paired-end sequencing with a read length of 150 bp on an Illumina NovaSeq X instrument.

***RNA***

Poly(A) RNA-Seq libraries were prepared using the NEBNext ^®^ Ultra™ II Directional RNA Library Prep Kit for Illumina (New England Biolabs), following the manufacturer's instructions. Poly(A) mRNA in the total RNA solution was isolated using oligo(dT) beads, converted to cDNA, and uniquely indexed; 14 PCR cycles were performed. Libraries were size-selected to produce fragments between 100–300 bp. Libraries were quantified, normalised, pooled to a final concentration of 2.8 nM, and diluted to 150 pM for loading. Sequencing was carried out on the the Illumina NovaSeq X instrument, generating paired-end reads. RNA data are publicly available under the same BioProject.

### Genome assembly, curation and evaluation

***Assembly***

Prior to assembly of the PacBio HiFi reads, a database of *k*-mer counts ( *k* = 31) was generated from the filtered reads using FastK. GenomeScope2 ( Ranallo-Benavidez *et al.*, 2020) was used to analyse the *k*-mer frequency distributions, providing estimates of genome size, heterozygosity, and repeat content.

The HiFi reads were assembled using Hifiasm in Hi-C phasing mode ( Cheng *et al.*, 2021; Cheng *et al.*, 2022), resulting in a pair of haplotype-resolved assemblies. The Hi-C reads were mapped to the primary contigs using bwa-mem2 ( Vasimuddin *et al.*, 2019). The contigs were further scaffolded using the provided Hi-C data ( Rao *et al.*, 2014) in YaHS ( Zhou *et al.*, 2023) using the --break option for handling potential misassemblies. The scaffolded assemblies were evaluated using Gfastats ( Formenti *et al.*, 2022), BUSCO ( Manni *et al.*, 2021) and MERQURY.FK ( Rhie *et al.*, 2020).

The mitochondrial genome was assembled using MitoHiFi ( Uliano-Silva *et al.*, 2023), which runs MitoFinder ( Allio *et al.*, 2020) and uses these annotations to select the final mitochondrial contig and to ensure the general quality of the sequence.

***Assembly curation***

The assembly was decontaminated using the Assembly Screen for Cobionts and Contaminants (ASCC) pipeline (article in preparation). Flat files and maps used in curation were generated in TreeVal ( Pointon *et al.*, 2023). Manual curation was primarily conducted using PretextView ( Harry, 2022), with additional insights provided by JBrowse2 ( Diesh *et al.*, 2023) and HiGlass ( Kerpedjiev *et al.*, 2018). Scaffolds were visually inspected and corrected as described by Howe *et al.* (2021). Any identified contamination, missed joins, and mis-joins were corrected, and duplicate sequences were tagged and removed. Sex chromosomes were identified by synteny analysis. The curation process is documented at https://gitlab.com/wtsi-grit/rapid-curation (article in preparation).

***Assembly quality assessment***

The Merqury.FK tool ( Rhie *et al.*, 2020), run in a Singularity container ( Kurtzer *et al.*, 2017), was used to evaluate *k*-mer completeness and assembly quality for the primary and alternate haplotypes using the *k*-mer databases ( *k* = 31) that were computed prior to genome assembly. The analysis outputs included assembly QV scores and completeness statistics.

A Hi-C contact map was produced for the final version of the assembly. The Hi-C reads were aligned using bwa-mem2 ( Vasimuddin *et al.*, 2019) and the alignment files were combined using SAMtools ( Danecek *et al.*, 2021). The Hi-C alignments were converted into a contact map using BEDTools ( Quinlan & Hall, 2010) and the Cooler tool suite ( Abdennur & Mirny, 2020). The contact map is visualised in HiGlass ( Kerpedjiev *et al.*, 2018).

The blobtoolkit pipeline is a Nextflow port of the previous Snakemake Blobtoolkit pipeline ( Challis *et al.*, 2020). It aligns the PacBio reads in SAMtools and minimap2 ( Li, 2018) and generates coverage tracks for regions of fixed size. In parallel, it queries the GoaT database ( Challis *et al.*, 2023) to identify all matching BUSCO lineages to run BUSCO ( Manni *et al.*, 2021). For the three domain-level BUSCO lineages, the pipeline aligns the BUSCO genes to the UniProt Reference Proteomes database ( Bateman *et al.*, 2023) with DIAMOND blastp ( Buchfink *et al.*, 2021). The genome is also divided into chunks according to the density of the BUSCO genes from the closest taxonomic lineage, and each chunk is aligned to the UniProt Reference Proteomes database using DIAMOND blastx. Genome sequences without a hit are chunked using seqtk and aligned to the NT database with blastn ( Altschul *et al.*, 1990). The blobtools suite combines all these outputs into a blobdir for visualisation.

The blobtoolkit pipeline was developed using nf-core tooling ( Ewels *et al.*, 2020) and MultiQC ( Ewels *et al.*, 2016), relying on the Conda package manager, the Bioconda initiative ( Grüning *et al.*, 2018), the Biocontainers infrastructure ( da Veiga Leprevost *et al.*, 2017), as well as the Docker ( Merkel, 2014) and Singularity ( Kurtzer *et al.*, 2017) containerisation solutions.

Table 4 contains a list of relevant software tool versions and sources.

**Table 4.** Software tools: versions and sources.

Software tool	Version	Source
BEDTools	2.30.0	https://github.com/arq5x/bedtools2
BLAST	2.14.0	ftp://ftp.ncbi.nlm.nih.gov/blast/executables/blast+/
BlobToolKit	4.3.9	https://github.com/blobtoolkit/blobtoolkit
BUSCO	5.5.0	https://gitlab.com/ezlab/busco
bwa-mem2	2.2.1	https://github.com/bwa-mem2/bwa-mem2
Cooler	0.8.11	https://github.com/open2c/cooler
DIAMOND	2.1.8	https://github.com/bbuchfink/diamond
fasta_windows	0.2.4	https://github.com/tolkit/fasta_windows
FastK	1.1	https://github.com/thegenemyers/FASTK
Gfastats	1.3.6	https://github.com/vgl-hub/gfastats
GoaT CLI	0.2.5	https://github.com/genomehubs/goat-cli
Hifiasm	0.19.8-r603	https://github.com/chhylp123/hifiasm
HiGlass	1.13.4	https://github.com/higlass/higlass
Merqury.FK	1.1.2	https://github.com/thegenemyers/MERQURY.FK
Minimap2	2.24-r1122	https://github.com/lh3/minimap2
MitoHiFi	3	https://github.com/marcelauliano/MitoHiFi
MultiQC	1.14, 1.17, and 1.18	https://github.com/MultiQC/MultiQC
Nextflow	23.10.0	https://github.com/nextflow-io/nextflow
PretextView	0.2.5	https://github.com/sanger-tol/PretextView
samtools	1.19.2	https://github.com/samtools/samtools
sanger-tol/ ascc	-	https://github.com/sanger-tol/ascc
sanger-tol/blobtoolkit	0.5.1	https://github.com/sanger-tol/blobtoolkit
Seqtk	1.3	https://github.com/lh3/seqtk
Singularity	3.9.0	https://github.com/sylabs/singularity
TreeVal	1.2.0	https://github.com/sanger-tol/treeval
YaHS	1.2a.2	https://github.com/c-zhou/yahs

### Wellcome Sanger Institute – Legal and Governance

The materials that have contributed to this genome note have been supplied by a Darwin Tree of Life Partner. The submission of materials by a Darwin Tree of Life Partner is subject to the **‘Darwin Tree of Life Project Sampling Code of Practice’**, which can be found in full on the Darwin Tree of Life website here. By agreeing with and signing up to the Sampling Code of Practice, the Darwin Tree of Life Partner agrees they will meet the legal and ethical requirements and standards set out within this document in respect of all samples acquired for, and supplied to, the Darwin Tree of Life Project.

Further, the Wellcome Sanger Institute employs a process whereby due diligence is carried out proportionate to the nature of the materials themselves, and the circumstances under which they have been/are to be collected and provided for use. The purpose of this is to address and mitigate any potential legal and/or ethical implications of receipt and use of the materials as part of the research project, and to ensure that in doing so we align with best practice wherever possible. The overarching areas of consideration are:

•    Ethical review of provenance and sourcing of the material

•    Legality of collection, transfer and use (national and international)

Each transfer of samples is further undertaken according to a Research Collaboration Agreement or Material Transfer Agreement entered into by the Darwin Tree of Life Partner, Genome Research Limited (operating as the Wellcome Sanger Institute), and in some circumstances other Darwin Tree of Life collaborators.

## Data Availability

European Nucleotide Archive: Buteo buteo (common buzzard). Accession number PRJEB74952; https://identifiers.org/ena.embl/PRJEB74952. The genome sequence is released openly for reuse. The *Buteo buteo* genome sequencing initiative is part of the Darwin Tree of Life (DToL) project. All raw sequence data and the assembly have been deposited in INSDC databases. The genome will be annotated using available RNA-Seq data and presented through the Ensembl pipeline at the European Bioinformatics Institute. Raw data and assembly accession identifiers are reported in Table 1 and Table 2.

## Author information

Members of the Natural History Museum Genome Acquisition Lab are listed here: https://doi.org/10.5281/zenodo.12159242.

Members of the Darwin Tree of Life Barcoding collective are listed here: https://doi.org/10.5281/zenodo.12158331.

Members of the Wellcome Sanger Institute Tree of Life Management, Samples and Laboratory team are listed here: https://doi.org/10.5281/zenodo.12162482.

Members of Wellcome Sanger Institute Scientific Operations: Sequencing Operations are listed here: https://doi.org/10.5281/zenodo.12165051.

Members of the Wellcome Sanger Institute Tree of Life Core Informatics team are listed here: https://doi.org/10.5281/zenodo.12160324.

Members of the Tree of Life Core Informatics collective are listed here: https://doi.org/10.5281/zenodo.12205391.

Members of the Darwin Tree of Life Consortium are listed here: https://doi.org/10.5281/zenodo.4783558.
