# Supplementary material for: The genome sequence of the Common Buzzard, Buteo buteo (Linnaeus, 1758)
Source: Wellcome Open Res. 2025 Sep 26;10:130. Originally published 2025 Mar 14. [Version 2] doi: 10.12688/wellcomeopenres.23873.2 (PMC12481152; doi:10.12688/wellcomeopenres.23873.2)
Supplement: Peer review history

# Reviewer report: Reviewer response for version 2

**DOI:** 10.21956/wellcomeopenres.27565.r134449

**Published:** 2025-09-29

**Authors:** Natalie J Forsdick

**Affiliations:** 1 Manaaki Whenua Landcare Research, Auckland, New Zealand

Thanks to the authors for addressing my previous concerns.

Are sufficient details of methods and materials provided to allow replication by others?

Yes

Is the rationale for creating the dataset(s) clearly described?

Yes

Are the datasets clearly presented in a useable and accessible format?

Yes

Are the protocols appropriate and is the work technically sound?

Yes

Reviewer Expertise:

Genome assembly; conservation genomics; population genomics

I confirm that I have read this submission and believe that I have an appropriate level of expertise to confirm that it is of an acceptable scientific standard.

# Reviewer report: Reviewer response for version 1

**DOI:** 10.21956/wellcomeopenres.26339.r120743

**Published:** 2025-04-09

**Authors:** Natalie J Forsdick

**Affiliations:** 1 Manaaki Whenua Landcare Research, Auckland, New Zealand

The manuscript 'The genome sequence of the Common Buzzard, *Buteo buteo *(Linnaeus, 1758)' clearly describes the sequencing and assembly of a genome for the Common Buzzard, using PacBio HiFi and Hi-C data. Background, Methods, and Results are clearly described. My primary concern is that no mention is made of animal ethics permitting for the lethal sampling of this individual. 

 Also, though I understand the RNA-seq data will be used for downstream annotation, it is not used here, and so inclusion of details pertaining to the RNA sequencing is superfluous here, and I recommend it only be included in subsequent publication using that data.

Are sufficient details of methods and materials provided to allow replication by others?

Yes

Is the rationale for creating the dataset(s) clearly described?

Yes

Are the datasets clearly presented in a useable and accessible format?

Yes

Are the protocols appropriate and is the work technically sound?

Yes

Reviewer Expertise:

Genome assembly; conservation genomics; population genomics

I confirm that I have read this submission and believe that I have an appropriate level of expertise to confirm that it is of an acceptable scientific standard, however I have significant reservations, as outlined above.

# Reviewer report: Reviewer response for version 1

**DOI:** 10.21956/wellcomeopenres.26339.r120739

**Published:** 2025-04-01

**Authors:** Chiara Bortoluzzi

**Affiliations:** 1 SIB Swiss Institute of Bioinformatics, Lausanne, Switzerland

The article discusses the genome assembly of a male *Buteo buteo *(common buzzard), a bird species that is widespread in the UK and has seen a population recovery in recent years due to legal protections, changes in land use, and reduced pesticide use. The article offers a thorough overview and description of the materials and methods used to generate the two haplotypes, which are made available to the community via INSDC and ENA. The article is well-written, providing sufficient information and details to ensure the work is reproducible. Additionally, the information is clearly presented in a user-friendly and accessible format.

 My only comment is that, contrary to what is stated in the Results section, the BUSCO completeness score for haplotype 2 is not reported (see Table 2), only for haplotype 1. While I understand that haplotype 2 is a scaffold-level assembly, either the completeness score for haplotype 2 should be included, or the sentence in the main text (see 'Assembly quality metrics' ) should be revised.

Are sufficient details of methods and materials provided to allow replication by others?

Yes

Is the rationale for creating the dataset(s) clearly described?

Yes

Are the datasets clearly presented in a useable and accessible format?

Yes

Are the protocols appropriate and is the work technically sound?

Yes

Reviewer Expertise:

Comparative genomics, population genomics, conservation genomics, data management

I confirm that I have read this submission and believe that I have an appropriate level of expertise to confirm that it is of an acceptable scientific standard.
